# Sex-related differences in markers of immune activation in virologically suppressed HIV-infected patients

**DOI:** 10.1186/s13293-020-00302-x

**Published:** 2020-05-01

**Authors:** Letizia Santinelli, Giancarlo Ceccarelli, Cristian Borrazzo, Giuseppe Pietro Innocenti, Federica Frasca, Eugenio Nelson Cavallari, Luigi Celani, Chiara Nonne, Claudio Maria Mastroianni, Gabriella d’Ettorre

**Affiliations:** 1 Department of Molecular Medicine, Sapienza University of Rome, Rome, Italy; 2 Department of Public Health and Infectious Diseases, Sapienza University of Rome, Rome, Italy

**Keywords:** HIV, Sex, Gut, PBMC, Immune activation

## Abstract

**Objectives:**

Gender-specific studies remain a neglected area of biomedical research. Recent reports have emphasized that sex-related biological factors may affect disease progression during HIV-1 infection. The aim of this study was to investigate the influence of sex on the levels of immune activation in the gut and in peripheral blood of individuals with HIV treated with fully suppressive antiretroviral therapy (ART).

**Methods:**

Thirty individuals with HIV undergoing long-term fully suppressive ART were enrolled in this study. Lamina propria lymphocytes (LPL) and peripheral blood mononuclear cells (PBMCs) were isolated from gut biopsies collected by pancolonoscopy and peripheral blood samples. The expression of markers of immune activation was evaluated by multi-parametric flow cytometry. This is a sub analysis of ClinicalTrials.gov Identifier: NCT02276326

**Results:**

We observed differences in the levels of immune activation in the gut and in PBMCs, with values higher in the gut compartment compared to PBMCs.

In addition, we found that the mean value of the levels of immune activation was higher in the women than in the men. Finally, we measured the markers of immune activation by mean relative difference (MRD) and confirmed the higher value in the women.

**Conclusion:**

A significant sex-related difference in the level of immune activation was observed in a population of individuals with HIV on long-term ART. A more complete characterization of these differences may support the introduction of sex-specific approaches in the clinical management of individuals with HIV.

## Introduction

In 2015, the National Institutes of Health (NIH) established that sex is a biological variable (SABV) to take into account for NIH funding and scientific publication [1]. This statement was based on scientific evidence that sex affects innate and adaptive immunity and results in sex-specific outcomes of autoimmune pathologies, malignancies, infectious diseases, and vaccines [2]. Indeed, a growing body of published studies illustrates that sex hormones play an important role in the regulation of the immune system by releasing cytokines involved in the proliferation, differentiation, and maturation of immune cells, therefore introducing the concept of immune dimorphism [3, 4].

In recent years, evidence has accumulated which demonstrates that the progression of inflammatory diseases, such as HIV infection, as well as the response to therapy, may be influenced by sex-related variables [2, 5, 6]. In particular, sex-related differences in the viral kinetics of HIV infection were described. Griesbeck et al. observed a different response to HIV during the acute infection, with women showing higher viral load and stronger antiviral response than men [5]. In contrast, several studies revealed lower viremia in the initial phase of chronic HIV infection in women as compared to men (over 40% less circulating HIV RNA than men) [7]. These differences in virus replication during the initial phases of HIV infection could contribute to sex-related differences in the level of HIV-DNA in PBMCs. Moreover, higher levels of CD8+ T cell activation were found in untreated women with HIV-1 as compared to men and independent of the levels of plasma viremia [8]. Also supporting the impact of sex-related variables in HIV disease progression is the observation that woman have a 1.6-fold higher risk of developing AIDS [9]. Finally, changes in the levels of soluble markers of immune activation were reported in woman and men with chronic HIV infection, thus suggesting a potential predictive role in terms of HIV disease outcome [10].

In light of this information, studying the impact of sex-linked variables on the outcome of HIV infection and the response to antiretroviral therapy (ART) may improve the management of HIV infection. The aim of this study was to ascertain whether the levels of immune activation in the gut and peripheral blood are influenced by the sex of ART-treated HIV-1 positive patients with undetectable viremia.

## Methods

The study was approved by the institutional review board (Ethics Committee of Umberto I General Hospital, Rome), and all participants signed written informed consent.

### Enrollment

Thirty patients living with HIV (PLWH) treated with ART and virologically suppressed were recruited at the Department of Public Health and Infectious Diseases of “Sapienza” University of Rome (Italy). The inclusion criteria used to enroll HIV-positive patients were as follows: (i) to have signed the informed consent, (ii) men or women at least 18 years of age, (iii) on active ART, (iv) with HIV-1 RNA < 37 copies/mL and CD4^+^ T counts > 400 cells/mm^3^. Exclusion criteria were (i) history of or current inflammatory diseases of the small or large intestine; (ii) diarrhea; (iii) any current, past, or systemic malignancy; (iv) pregnancy.

This is a sub analysis of study identified as NCT02276326, registered on the ClinicalTrials.gov archive.

### T-cell phenotyping by flow cytometry

#### Sample collection and cell isolation

All individuals underwent a total colonoscopy and retrograde ileoscopy for at least 10 cm of distal ileum with conventional or slim scope (model CF or PCF-160 AI, Olympus Medical Europe GmbH, Hamburg, Germany). Specimens (two biopsies from each site) from the terminal ileum, cecum, ascending, transverse, and descending colon were obtained. Immune activation was evaluated in 60 biological samples (30 PBMCs and 30 gut biopsies). Moreover, the blood sample collection by venepuncture was performed in all subjects enrolled: whole blood was collected in vacutainer tubes containing ethylenediaminetetraacetic acid (BD Biosciences, San Jose, CA), and PBMC were separated by Ficoll gradient centrifugation (Lympholyte, Cedarlane Labs, Hornby, Ontario, Canada), followed by two washes in phosphate-buffered saline. PBMCs were used for immune phenotyping or cultured overnight for stimulation. Biopsies from intestinal sites were mixed with each other and processed. Briefly, biopsies collected in RPMI 1640 (heat inactivated 10% fetal bovine serum) were washed twice with EDTA wash media, resuspended, and incubated for 1 h at room temperature in EDTA solution 5 mM on automatic shaker. Supernatant containing intraepithelial lymphocytes was removed, and biopsies were digested by 1-h incubation at 37 °C in pre-warmed RPMI 1640 (heat inactivated 10% fetal bovine serum) with 1 mg/mL collagenase (Sigma–Aldrich, Milan, Italy) and 1.5 U DNAse I (Sigma–Aldrich), bringing to the isolation of LPL, then filtered through a 70 μm cellular strainer (Becton Dickinson).

#### Surface marker staining

PBMC and LPL were aliquoted in 1 × 10^6^ cells/mL and 250 × 10^3^ respectively with RPMI medium plus 10% FBS (fetal bovine serum). Cells were stained with the specific mAbs, previously described, at dilution recommended by the manufacturer and incubated in the dark for 10 min at 4 °C. The following anti-human monoclonal antibodies were added: CD3-PerCP, CD4-APC-Vio770, CD8-FITC, CD45RO-PEVio770, CD27-VioBlue, CD38-APC, and HLA-DR-PE (Miltenyi Biotec, Bergisch Gladbach, Germany). The expression of markers of immune activation (CD38, HLADR) on naïve, central memory (CMEM), and effector memory (TEM) CD4 and CD8 T-cells was evaluated by multi-parametric flow cytometry (Table 1). Stained cells were previously washed in PBS, were acquired, and analyzed by Miltenyi Biotec flow cytometer-MACSQuant Analyzer (8 fluorescence channels, 3 lasers). Gating strategy and data analysis were conducted using the MACSQuantify software 2.5 (Miltenyi Biotec). At least 100,000 and 10,000 events in the CD3+ lymphocytes gate were analyzed for PBMCs and LPLs, respectively. Isotype controls were used as negative controls to differentiate non-specific background signal from specific antibody signal of CD38, HLA-DR markers.

**Table 1:** CD4 and CD8 T-cell immunophenotyping and their biological functions

	**Function**
HLA-DR	Critical for efficient antigen presentation to CD4+ T cells. HLA DR is expressed primarily by antigen presenting cells and, together with CD38, is a useful marker of T-cell activation following viral infection.
CD38	Multifunctional ecto-enzyme involved in signal transduction, cell adhesion, and calcium signaling. Used to study the processes of B- and T-cell differentiation and activation. Increased expression of CD38 on both CD4+ and CD8+ T-cells in HIV-infected patients is associated with disease progression.
CD27	Important for the generation and long-term maintenance of immune response. Memory marker.
CD45RO	Regulator of T-cell antigen signaling. Memory marker.
CD27^+^CD45RO^-^	Naïve cells recognize cognate antigen and initiate an immune response.
CD27^+^CD45RO^+^	Central memory (CM) T-cells are activated in secondary lymphoid organs following recognition of antigen on DCs and generate large numbers of effector cells.
CD27^−^CD45RO^+^	Effector memory (EM) cells exhibit effector function immediately upon recognition of antigen presented on non-professional APCs and limit the early spread of infection.

#### Cells activation and intracellular marker staining

In order to evaluate intracellular IL-17 production, both PBMC and LPL were seeded with RPMI medium plus 10% FBS (fetal bovine serum) at 2 × 10^6^ cells/mL and 1.5 × 10^6^ cells/mL respectively. Cells were activated with ionomycin (1 μg/mL, Sigma–Aldrich) and phorbol myristate acetate (PMA) (3 ng/mL, Sigma–Aldrich) in presence of BD GolgiStop (Becton Dickinson).

Cells were incubated overnight at 37 °C and 5% CO_2_ and then harvested, permeabilized, and stained.

The following anti-human monoclonal antibodies were used: CD3-PerCP, CD4-APC-Vio770, CD8-FITC, CD45RO-PEVio770, CD27-VioBlue, IL-17-PE. Samples were acquired by Miltenyi Biotec flow cytometer-MACSQuant Analyzer (8 fluorescence channels, 3 lasers). Gating strategy and data analysis were conducted using the MACSQuantify software 2.5 (Miltenyi Biotec).

#### Virological analysis

HIV-1 RNA copy numbers were evaluated in plasma samples collected from whole blood obtained in EDTA-containing tubes and stored at −80 °C. Combined with Siemens Healthcare’s nucleic acid extraction technology, a HIV-specific quantitative reverse polymerase chain reaction (Versant kPCR by Siemens Healthcare Diagnostic Inc., Tarrytown, NY) was used to measure the levels of HIV-RNA. The detection limit is 37 copies/ mL [11].

#### Statistical analysis

All data were analyzed, and all graphs were generated using MATLAB version 7.9.0.529 (R2009b). The demographic characteristics of HIV-1 patients were compared using Mann–Whitney test. All measurements were taken as mean and standard error or median and range (IQR 75th percentile, 25th percentile). The mean relative difference (MRD) is defined as

1 $$ MRD=\frac{\Delta  }{X_2}=\frac{X_1-{X}_2}{X_2} $$

where *X*_1_ is the measure of the peripheral blood marker, while *X*_2_ is the measure of the marker referred to the gut. For values greater than the reference to the gut value, the MRD should be a positive number (*D* > 0) and for values that are smaller, the MRD should be negative (*D* < 0). The MRD with 1 sigma is uncertain on the meaning for each marker. The same test was also used to compare HIV-1-positive patients divided into two groups male and female, and to compare gene expression levels measured in PBMC and gut collected from patients with HIV-1. To assess the distribution, the Kolmogorov-Smirnov test and histograms were used. The same tests with group differences were tested using Student *t* test or Mann–Whitney test for normally and non-normally distributed variables, respectively. The Fisher’s exact tests or Chi-square tests were used as appropriate to test group differences of proportions. Univariable analyses were performed to determine which variables were significantly associated with sex on the HIV patients. Unstandardized mean difference (USMD) and their 95% CIs were analyzed [12]. USMD has been computed as the difference between the follow-up in the men and women group, divided by the whole population variance. In all tests, the level of statistical significance was 0.05.

Finally, all potential confounders were entered a regression model based on prior knowledge or expected clinical relevance. Analyses were performed with a stepwise forward regression model, in which each variable with a *p* value < 0.05 (based on univariate analysis) was entered the model; all regression models included age and gender at initial evaluation, as covariates.

## Results

### Demographic and clinical characteristics of HIV-1-positive patients

The study population (*n* = 30) was composed of 15 men and 15 women, with an average age of 47 (± 8.7) years. Demographic and clinical characteristics of HIV-1-infected, stratified by sex, are shown in Table 2. They were started on ART during chronic HIV-1 infection with a median CD4 T-cell count of 300 at the beginning of therapy (IQR 115-377). At enrollment, all subjects had been virologically suppressed (< 37 HIV-1 RNA copies/mL) for at least 1 year, had a median CD4 T-cell count of 773 cells/mm^3^ (IQR 623-950 cells/mm^3^), and had received therapy for a median of 11 years (IQR 5-19). Stratifying the study population by gender, men showed a mean age of 43 years (±10), a median duration of therapy of 6.5 years (IQR 1-17), and a median CD4-Tcell count at enrollment of 667 (IQR 621–912), whereas women showed a mean age of 49 years (± 5), a median duration of therapy of 14 years (IQR 11–23) and a median CD4 T-cell count at enrollment of 789 cells/mm^3^ (IQR 708-918). No statistically significant differences between men and women were found in terms of demographics or clinical characteristics. Interestingly, the women enrolled had all reached menopause (clinically defined as “permanent cessation of menstruation occurred after 12 consecutive months of amenorrhea, for which there is no other obvious pathologic or physiologic cause”) [13].

**Table 2:** Demographic and clinical characteristics of HIV-1-infected

**Parameters**	**All population**			**Female**			**Male**			***p*value**
	**Mean ± SD**	**Median with IQR**	***n*(n%)**	**Mean ± SD**	**Median with IQR**	***n*(n%)**	**Mean ± SD**	**Median with IQR**	***n*(n%**)	
**Age (years)**	45 ± 8	47(44–52)	-	49 ± 5	51(45–52)	-	43 ± 9	46 (37–48)	-	0.067
**Gender (female)**	-	-	15 (50)	-	-	-	-	-	-	-
**Smokers**	-	-	17 (57)	-	-	6 (40)	-	-	11 (73)	0.762
**BMI (Kg/m2)**	23 ± 3	23 (21–25)		22 ± 4	21(19–24)		23 ± 2	24(22–25)	-	0.373
**Race (**Caucasian**)**	-	-	29 (97)	-		14 (93)	-	-	15 (100)	0.987
**Years on ART (years)**	12 ± 9	11 (5–19)	-	17 ± 6	15(11–22)	-	9 ± 9	5(1–15)	-	**0.012**
**Years HIV diagnosis (years)**	12 ± 9	11(5–19)	-	17 ± 6	15(11–22)	-	9 ± 9	5(1–15)	-	**0.014**
**CD4+ actual (cells/mm**^**3**^**)**	744 ± 299	708 (623–886)	-	790 ± 275	789 (692–886)	-	718 ± 317	667 (623–825)	-	0.560
**CD4+ (%)**	33 ± 11	34 (30–38)	-	38 ± 5	36 (30–38)	-	30 ± 12	32 (24–37)	-	**0.032**
**CD4+ nadir (cells/mm**^**3**^**)**	283 ± 223	301 (74–382)	-	182 ± 144	163 (74–278)	-	321 ± 239	322 (109–522)		0.117
**HCV infection**	-	-	3 (10)	-	-	1 (7)	-	-	2 (13)	0.922
**HIV-RNA actual (copies/mL)**	< 37	-	-	< 37	-	-	<37	-	-	1.000

*SD* standard deviation, *BMI* body mass index, *ART* antiretroviral therapy

### Levels of markers of immune activation in the gut and PBMCs

**Fig. 1:**
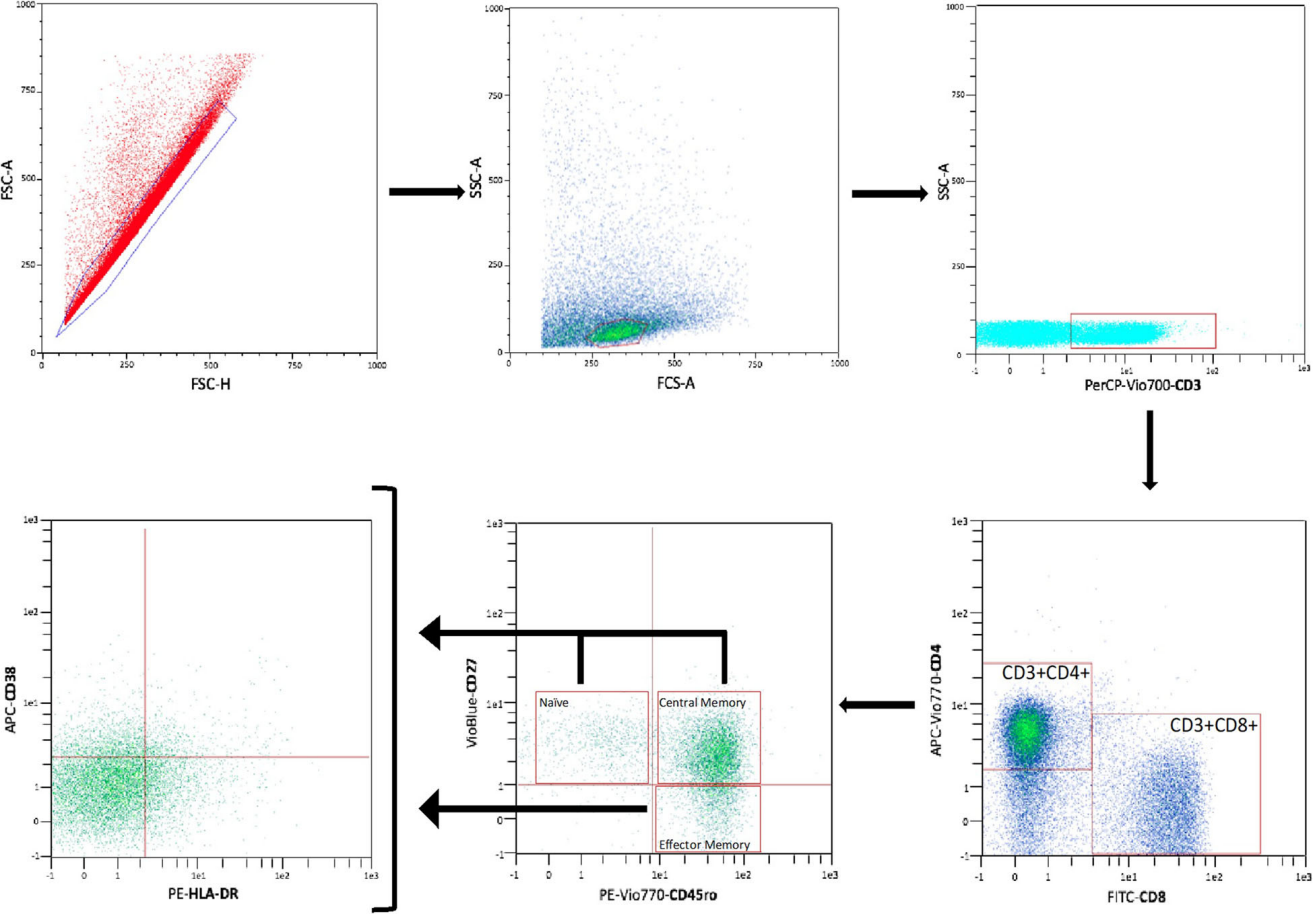
*Representative flow* cytometry *plots* illustrating the gating strategy used for analysis of PBMC and LPL samples

We measured 36 markers of immune activation expressed on CD4+ and CD8+ T cell in the gut and in PBMCs both in men and in women and found that for 23 markers the values were significantly higher in the gut than in PBMCs (Table 3). *Representative flow* cytometry *plots,* illustrating the gating strategy used for PBMC and LPL analysis, are shown in Fig. 1. When we considered these markers of immune activation expressed on CD4+ and CD8+ T cell only in the men, we observed that 33 markers of immune activation were higher in the gut compared to PBMCs, but only 19 markers showed a statistically significant difference as showed in Table 3. In the group of women, 34 markers were higher in the gut with respect to the PBMCs, and among these, 25 showed a statistically significant difference (Table 3).

**Table 3:** Differences in immune-activation markers between the gut and PBMC

	**All (PBMC)**	**All (GUT)**		**Male (PBMC)**	**Male (GUT)**		**Female (PBMC)**	**Female (GUT)**	
**Markers**	**Mean ± SE**	**Mean ± SE**	***p*value**	**Mean ± SE**	**Mean ± SE**	***p*value**	**Mean ± SE**	**Mean ± SE**	***p*value**
**CD4**^**+**^**naïve CD38**^**+**^	15.82 ± 4.07	18.92 ± 3.70	0.575	7.49 ± 1.25	17.79 ± 3.02	**0.006**	25.08 ± 4.97	20.18 ± 4.51	0.472
**CD4**^**+**^**naïve CD38**^**+**^**HLA-DR**^**+**^	1.75 ± 0.70	8.97 ± 1.92	**0.001**	0.54 ± 0.15	9.59 ± 1.68	**0.001**	3.10 ± 0.88	8.28 ± 2.26	**0.047**
**CD4**^**+**^**naïve HLA-DR**^**+**^	4.97 ± 2.21	17.21 ± 2.45	**0.001**	2.56 ± 0.46	17.94 ± 2.14	**0.001**	7.65 ± 2.95	16.39 ± 2.87	**0.044**
**CD4**^**+**^**TCM CD38**^**+**^	10.89 ± 2.71	12.69 ± 2.51	0.628	10.14 ± 0.77	14.17 ± 2.24	0.108	11.72 ± 3.75	11.05 ± 2.85	0.889
**CD4**^**+**^**TCM CD38**^**+**^**HLA-DR+**	1.47 ± 0.23	5.89 ± 1.67	**0.014**	1.5 ± 0.20	8.78 ± 1.99	**0.003**	1.44 ± 0.59	2.68 ± 0.63	0.164
**CD4**^**+**^**TCM HLA-DR+**	2.94 ± 2.88	13.60 ± 2.89	**0.012**	7.34 ± 2.16	14.25 ± 2.15	**0.032**	6.12 ± 1.17	12.88 ± 3.68	0.100
**CD4**^**+**^**TEM CD38**^**+**^	4.35 ± 0.70	10.50 ± 2.03	**0.007**	4.65 ± 0.68	11.03 ± 1.88	**0.005**	4.02 ± 0.83	9.91 ± 2.29	**0.028**
**CD4**^**+**^**TEM CD38**^**+**^**HLA-DR**^**+**^	2.30 ± 0.30	6.02 ± 1.32	**0.010**	2.60 ± 0.32	6.26 ± 1.56	**0.036**	1.96 ± 0.44	5.74 ± 1.08	**0.005**
**CD4**^**+**^**TEM HLA-DR**^**+**^	11.96 ± 1.26	20.18 ± 2.43	**0.005**	11.52 ± 1.08	16.94 ± 2.23	**0.041**	12.46 ± 1.77	23.77 ± 2.43	**0.001**
**CD8**^**+**^**naïve CD38**^**+**^	12.45 ± 3.31	7.71 ± 1.92	0.223	5.99 ± 0.76	7.60 ± 1.93	0.448	19.63 ± 3.94	7.83 ± 2.02	**0.015**
**CD8**^**+**^**naïve CD38**^**+**^**HLA-DR**^**+**^	1.36 ± 0.35	6.85 ± 1.71	**0.004**	0.76 ± 0.15	5.18 ± 1.26	**0.004**	2.02 ± 0.43	8.72 ± 2.06	**0.006**
**CD8**^**+**^**naïve HLA-DR**^**+**^	4.38 ± 0.77	21.68 ± 3.87	**0.001**	3.75 ± 0.39	17.33 ± 1.57	**0.001**	5.07 ± 1.01	26.52 ± 5.25	**0.001**
**CD8**^**+**^**TCM CD38**^**+**^	6.13 ± 2.10	9.72 ± 2.16	0.240	4.30 ± 0.79	10.42 ± 2.07	**0.013**	8.16 ± 2.79	8.94 ± 2.36	0.834
**CD8**^**+**^**TCM CD38**^**+**^**HLA-DR+**	4.09 ± 0.69	9.32 ± 2.22	**0.032**	5.10 ± 0.76	9.83 ± 2.26	0.064	2.96 ± 0.48	8.74 ± 2.31	**0.027**
**CD8**^**+**^**TCM HLA-DR+**	8.54 ± 1.51	20.23 ± 3.88	**0.008**	8.81 ± 1.58	18.52 ± 1.77	**0.001**	8.24 ± 1.45	22.14 ± 5.47	**0.027**
**CD8**^**+**^**TEM CD38**^**+**^	6.50 ± 2.56	14.77 ± 2.64	**0.029**	3.74 ± 0.40	15.75 ± 2.56	**0.001**	9.57 ± 3.49	13.67 ± 2.84	0.372
**CD8**^**+**^**TEM CD38**^**+**^**HLA-DR**^**+**^	4.25 ± 0.58	13.33 ± 2.89	**0.004**	4.11 ± 0.57	12.36 ± 3.10	**0.020**	4.41 ± 0.60	14.40 ± 2.81	**0.003**
**CD8**^**+**^**TEM HLA-DR**^**+**^	11.40 ± 1.27	19.88 ± 3.55	**0.031**	10.82 ± 1.24	15.08 ± 1.89	0.072	12.05 ± 1.52	25.21 ± 4.50	**0.013**
**CD4**^**+**^**naïve Th1**	1.84 ± 0.81	5.26 ± 2.05	0.129	2.21 ± 1.10	2.09 ± 0.87	0.933	1.42 ± 0.33	8.78 ± 2.61	**0.014**
**CD4**^**+**^**naïve Th17**	0.01 ± 0.01	0.18 ± 0.07	**0.030**	0.01 ± 0.00	0.06 ± 0.04	0.203	0.01 ± 0.01	0.32 ± 0.09	**0.005**
**CD4**^**+**^**naïve Th1 Th17**	0.13 ± 0.02	2.37 ± 0.67	**0.003**	0.13 ± 0.03	1.82 ± 0.66	**0.023**	0.14 ± 0.02	2.97 ± 0.69	**0.001**
**CD4**^**+**^**TCM Th1**	10.87 ± 3.41	20.77 ± 4.50	0.086	9.38 ± 3.93	12.31 ± 3.11	0.564	12.53 ± 2.91	30.17 ± 4.70	**0.004**
**CD4**^**+**^**TCM Th17**	0.06 ± 0.02	1.57 ± 0.47	**0.004**	0.03 ± 0.01	0.92 ± 0.34	**0.023**	0.09 ± 0.03	2.30 ± 0.55	**0.001**
**CD4**^**+**^**TCM Th1 Th17**	0.46 ± 0.11	3.20 ± 1.08	**0.018**	0.38 ± 0.10	1.32 ± 0.32	**0.015**	0.55 ± 0.13	5.29 ± 1.38	**0.004**
**CD4**^**+**^**TEM Th1**	15.03 ± 4.39	18.79 ± 4.82	0.567	10.92 ± 4.67	7.34 ± 2.05	0.491	19.59 ± 4.00	31.51 ± 4.95	0.073
**CD4**^**+**^**TEM Th17**	0.13 ± 0.04	1.94 ± 0.67	**0.012**	0.07 ± 0.03	0.56 ± 0.20	**0.039**	0.2 ± 0.05	3.49 ± 0.80	**0.001**
**CD4**^**+**^**TEM Th1 Th17**	0.49 ± 0.13	3.54 ± 1.34	**0.032**	0.26 ± 0.06	0.93 ± 0.33	0.073	0.74 ± 0.16	6.43 ± 1.65	**0.004**
**CD8**^**+**^**naïve Tc1**	7.99 ± 2.70	21.18 ± 6.03	0.053	5.87 ± 2.65	10.75 ± 3.33	0.263	10.33 ± 2.77	32.76 ± 7.09	**0.009**
**CD8**^**+**^**naïve Tc17**	0.04 ± 0.03	0.27 ± 0.10	**0.045**	0.01 ± 0.00	0.10 ± 0.03	0.053	0.07 ± 0.04	0.46 ± 0.13	**0.014**
**CD8**^**+**^**naïve Tc1 Tc17**	0.15 ± 0.05	0.99 ± 0.42	**0.057**	0.09 ± 0.02	0.51 ± 0.16	**0.025**	0.22 ± 0.07	1.53 ± 0.57	**0.040**
**CD8**^**+**^**TCM Tc1**	26.81 ± 7.59	32.40 ± 6.84	0.587	19.82 ± 7.73	21.59 ± 5.05	0.849	34.58 ± 7.33	44.41 ± 7.48	0.357
**CD8**^**+**^**TCM Tc17**	0.14 ± 0.07	0.36 ± 0.12	0.135	0.08 ± 0.03	0.21 ± 0.09	0.216	0.21 ± 0.10	0.53 ± 0.14	0.081
**CD8**^**+**^**TCM Tc1 Tc17**	0.26 ± 0.12	0.51 ± 0.14	0.220	0.13 ± 0.04	0.33 ± 0.11	0.136	0.42 ± 0.17	0.71 ± 0.16	0.240
**CD8**^**+**^**TEM Tc1**	26.30 ± 7.46	30.65 ± 7.30	0.678	18.13 ± 7.81	13.99 ± 3.61	0.636	35.37 ± 6.70	49.15 ± 7.52	0.183
**CD8**^**+**^**TEM Tc17**	0.03 ± 0.01	0.29 ± 0.14	0.084	0.01 ± 0.00	0.12 ± 0.10	0.301	0.06 ± 0.01	0.47 ± 0.17	**0.028**
**CD8**^**+**^**TEM Tc1 Tc17**	0.07 ± 0.03	0.44 ± 0.15	**0.026**	0.03 ± 0.00	0.30 ± 0.13	0.055	0.12 ± 0.04	0.59 ± 0.17	**0.019**

Columns show marker name, mean ± sigma values of each marker expressed in the gut and PBMC of all patients, men and women, *p* value. The statistical significance is achieved when *p* < 0.05^†^. Statistical differences between the gut and PBMC were evaluated using Student’s *t* tests

### Differences in immune activation markers: impact of sex

Overall, in our study population, the levels of immune activation were higher in the gut than in the PBMCs (MRD value − 1.12 ± 0.67).

**Fig. 2:**
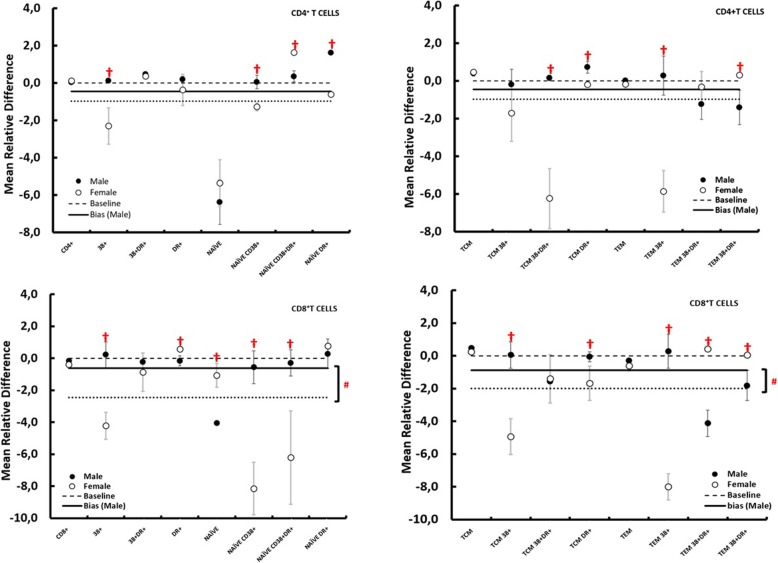
Differences in immune-activation markers between men and women. Ranked mean relative differences (MRD) for male (black-filled circle) and female (gray open circle) for each marker. Vertical bars indicate the errors. The errors on the MRD are calculated with standard error propagation. The same axis value ranges were used in each case for ease of comparison. The dotted line markers the case of zero difference between the marker measurement in the peripheral blood and in the GUT. *P* values are significant: bias male vs. bias female (#) *P* < 0.05; immune activation markers: man vs. women (†) *P* < 0.05

When we analyzed activated immune cell types by sex, we observed statistically significant differences between men and women as reported in Table 4. Interestingly, ranked MRDs in women are often below the 0 line; hence, on average, the marker has a higher value in the gut than the peripheral blood, while in men the MRDs are on average equal to 0 (Fig. 2). A significant difference in the immunological response linked to sex was also highlighted by the lower average MRD value calculated for all markers in men (− 0.52 ± 0.42) than in women (− 1.72 ± 0.65).

**Table 4:** Differences in immune-activation markers between men and women

	**Markers**	**Men** **[MRD ± sigma]**	**Women** **[MRD ± sigma]**	***p*-value**
**CD4+**	**CD4+**	0.03 ± 0.09	0.11 ± 0.06	0.07
	**CD4+ 38+**	0.11 ± 0.10	-2.29 ± 0.97	**0.024†**
	**CD4+ 38+DR+**	0.47 ± 0.10	0.36 ± 0.13	0.053
	**CD4+ DR+**	0.19 ± 0.16	-0.37 ± 0.83	0.052
	**CD4+ NAÏVE**	-6.38 ± 1.19	-5.36 ± 1.24	0.072
	**CD4+ NAÏVE CD38+**	0.05 ± 0.35	-1.28 ± 0.21	**0.044†**
	**CD4+ NAÏVE**	0.35 ± 0.30	1.62 ± 0.09	**0.044†**
	**CD38+DR+**	1.61 ± 0.06	-0.61 ± 0.06	**0.010†**
	**CD4+ NAÏVE DR+**	0.40 ± 0.06	0.45 ± 0.05	0.487
	**CD4+ TCM**	-0.19 ± 0.14	-1.72 ± 1.49	0.324
	**CD4+ TCM 38+**	0.16 ± 0.20	-6.23 ± 1.59	**0.035†**
	**CD4+ TCM 38+DR+**	0.72 ± 1.17	-0.19 ± 0.12	**0.047†**
	**CD4+ TCM DR+**	0.01 ± 0.18	-0.19 ± 0.24	0.523
	**CD4+ TEM**	0.28 ± 0.19	-5.86 ± 1.11	**0.033†**
	**CD4+ TEM 38+**	-1.23 ± 0.55	-0.32 ± 0.81	0.370
	**CD4+ TEM 38+DR+**	-1.40 ± 0.52	0.31 ± 0.11	**0.007†**
	**CD4+ TEM DR+**	-0.15 ± 0.08	-0.38 ± 0.11	0.094
	**CD8+**	0.23 ± 0.17	-4.22 ± 0.84	**0.027†**
**CD8**^**+**^	**CD8+ 38+**	-0.21 ± 0.24	-0.86 ± 1.19	0.060
	**CD8+ 38+DR+**	-0.16 ± 0.23	0.59 ± 0.08	**0.010†**
	**CD8+ DR+**	-4.05 ± 1.51	-1.06 ± 0.74	**0.010†**
	**CD8+ NAÏVE**	-0.56 ± 0.34	-8.14 ± 1.64	**0.024†**
	**CD8+ NAÏVE CD38+**	-0.29 ± 0.61	-6.21 ± 2.91	**0.041†**
	**CD8+ NAÏVE**	0.29 ± 0.08	0.76 ± 0.04	0.103
	**CD38+DR+**	0.48 ± 0.08	0.26 ± 0.02	0.243
	**CD8+ NAÏVE DR+**	0.05 ± 0.29	-4.92 ± 1.09	**0.024†**
	**CD8+ TCM**	-1.55 ± 0.60	-1.39 ± 1.48	0.923
	**CD8+ TCM 38+**	-0.05 ± 0.18	-1.68 ± 1.06	**0.044†**
	**CD8+ TCM 38+DR+**	-0.29 ± 0.19	-0.61 ± 0.30	0.081
	**CD8+ TCM DR+**	0.28 ± 0.21	-7.99 ± 0.80	**0.032†**
	**CD8+ TEM**	-4.12 ± 2.49	0.43 ± 0.02	**0.010†**
	**CD8+ TEM 3**	-1.81 ± 0.87	0.06 ± 0.04	**0.007†**

Columns show (1) marker name, (2) men and (3) women MRD ± sigma, and (4) *p* value. The statistical significance is achieved when *p* < 0.05^†^. Data are expressed as mean ± sigma. Statistical differences between men and women patients were evaluated using Student’s *t* tests

Finally, the sensitivity analyses, stratified by sex, looking at the relationship between the immune cell levels and duration of ART therapy (univariate analysis) is reported in Table 2. On the other hand, the multivariate analysis, stratified by sex, with duration of ART therapy along with potentially other HIV-related variables is presented in Table 5.

**Table 5:** Multivariate analysis of factors associated with HIV-1-infection

**Parameters**	**Female**	**Male**	**Multivariate analysis**	
	***n*(n%)**	***n* (n%)**	**OR (95%CI)**	***p*value (+)**
Age (< 45 years)	2 (13)	5 (33)	0.3 (0.1–1.9)	0.208
Smokers	7 (47)	11 (73)	0.7 (0.2–2.7)	0.629
Years on ART (> 3 years)	9 (60)	10 (67)	0.81 (0.2–2.8)	0.752
Years HIV diagnosis (> 3 years)	9 (60)	10 (67)	0.81 (0.2–2.8)	0.752
CD4 NADIR				
< 200 cells/mm^3^	3 (20)	6 (40)	0.4 (0.1–1.9)	0.239
200–500 cells/mm^3^	3 (20)	5 (33)	0.5 (0.1–2.6)	0.413
> 500 cells/mm^3^	9 (60)	4 (27)	4.1 (0.9–19.2)	0.071
HCV infection	1 (7)	2 (13)	0.46 (0.1–5.7)	0.550

(+)Statistical significance shown if 95% Cl does not include 1

*OR* odds ratio, *95% CI* 95% Confidence level, *HCV* hepatitis C virus

## Discussion

*While sex- and gender*-specific *research remains a relatively* neglected topic in contemporary biomedical research*, recent* studies suggest that sex-related biological factors affect a number of physiological and pathological conditions. Under non-pathological conditions, X-chromosome, encoding for several genes involved in the immune regulation mechanisms, can potentially influence the immunocompetence. Moreover, steroid hormones play a pivotal role in immune modulation processes: in particular, estrogen receptors (ER) α and β are ubiquitously expressed by immune cells and are involved with estrogen at different stages of maturation of immune cells and regulation of immune responses [14]. Sex-related differences in the activation status of the immune system were reported also in HIV-negative population affected by chronic pathologies: for example, from a general point of view, autoimmune diseases are more prevalent in women than in men and diseases course, severity, and survival may also differ by sex. Accumulating evidences suggest that if genetic, epigenetic, and environmental factors contribute to those sex-related differences, sex hormones play probably a pivotal role: in fact, estrogens would seem to represent a powerful stimulus towards autoimmunity while androgens would have a protective role [15]. In autoimmune diseases, men are known to respond to infections with a prevalent Th1 response, whereas women show a Th2 predominant immune response and an increased antibody production [16]. For example, a distinct sexual dimorphism was observed in the immune activation status of patients with systemic lupus erythematosus and ankylosing spondylitis, particularly in the Th17 axis [17, 18].

As previously reported, HIV infection on treatment can be assimilated to a chronic inflammatory disease and chronic inflammation and persistence of the state of immune activation can be considered hallmarks of HIV infection, also if successfully ART treated.

Natural history of HIV infection is characterized by a progressive dysfunction of cellular and immune responses and a disruption of the gut mucosal barrier with significant impairment of mucosal immune defense, mainly including CD4+ T cells and Th17 cells. The damage of mucosal lymphoid tissue gut and barrier contributes, with gut microbiome dysbiosis, to microbial translocation and takes part in persistent systemic activation of immunity. Chronic immune activation is considered a predictor of HIV disease progression and is mainly characterized by increased levels of type I interferons (TFNs), proinflammatory cytokines, and activation markers (including CD38 and HLA-DR) on both CD4+ and CD8+ T cells [19].

The availability of ART has dramatically decreased the risk for AIDS-related pathologies changing the natural course of HIV infection. Despite the successful HIV suppression achieved with ART and the related meaningful benefits, some significant limitations of therapy are progressively emerging: the persistence of the generalized state of chronic immune activation and inflammation, linked to serious non-AIDS, events are among the most significant [20]. These conditions are the result of several factors mainly including dysbiosis, impairment of the gut mucosa and local and general immunity, persistent antigen stimulation due to microbial translocation and low residual viremia, co-infection-related damages, and cumulative ART toxicity [21].

Similarly to what has already been reported for HIV-negative patients, sex represents a significant variable capable of impacting also in the setting of HIV disease. Significant differences in the clinical history of HIV infection have been reported between females and males, with women experiencing an increased risk of developing AIDS compared to men for similar level of HIV-RNA and lower baseline viral load in primary infection. Despite sex specific differences in HIV, pathogenesis are yet poorly understood, and devoted studies are lacking; to explain this fact, it has been hypothesized that a less intense immune activation in HIV-infected males than in females could explain why men progress to AIDS at the same rate as or slower than women. In fact, some preliminary studies have observed that the greatest production of IFN-α by dendritic cells in response to HIV stimulation, observed in women, is associated to progesterone levels. At the same time, the intensity of immune activation seems to be directly related with IFN-α levels, which, in turn, are a risk factor for the HIV progression regardless of the viral load in chronic stage of HIV infection [14, 22–25].

In light of these preliminary evidences, we investigated possible sex-specific differences in immune activation markers on T-cells derived from peripheral blood and intestinal mucosa. All subjects enrolled in the analysis had undetectable plasma viremia, relatively high CD4 T-cell counts, and no differences in terms of clinical presentation and previous antiretroviral regimen were noted between men and women. Overall, in our study, the levels of immune activation were higher in the gut than in the PBMCs. However, when we analyzed by sex, we observed that immune activation markers expressed on CD4+ and CD8+ T cells were statistically significantly higher in women than in men both in the gut and in PBMCs. Moreover, *women showed more impressive alterations in the gut mucosal T-cell repertoire, especially in the Th1, Th17, and Th1/Th17 cell subsets* with *central* or *effector* memory *phenotype, compared to blood district than their men counterpart.* Functionally, these female-specific cell subset alterations might explain gender differences described in clinical presentation and outcomes of HIV infection [26, 27] and confirm previous observations showing that IL-17–expressing T-cell subset frequencies in PBMC *differ* from those found in the gut [27, 28]. Sex-related differences in hormonal setting probably play a pivotal role in this context with estrogen levels able to modulate CD4+ and CD8+ T cells subsets including Th1, Th2, Th17, and to influence several cytokines (TNF-α, IL-12, IL-6, IL-1β, IL-21) strictly related to the Th1/Tc1 and Th17/Tc17 response [29–34]. Taken together, these data might suggest that the gut T-cell *environment* could be a more sensitive site for HIV infection in *women* than in men, considering that both Th1/Th17 cells represent a preferred site for HIV-DNA long-term persistence and human lamina propria CD4+ T cells are naturally permissive to HIV-1 infection [35, 36].

The possible biases, represented by the higher duration of ART therapy observed in women enrolled along with potentially other HIV-related variables, were considered in a multivariate modeling: analysis showed that in our study sex remained as an independent predictor of difference in immune cell levels in the gut and periphery.

Interestingly, in our study, all women showed signs of premature aging (i.e., early menopause and lower levels of bone mineral density). Our hypothesis is that the persistence of higher levels of markers of immune activation in the gut of women, compared to men, may drive an accelerated senescence and suggest a different response to ART despite complete virological suppression. In this regard, a recent study reported the sex-related differences in immune activation and inflammatory markers observed in HIV-1-positive women and men after ART initiation [8]. These authors found higher levels of TNF and IFNγ in the women compared to the men after 48 weeks of follow-up, thus confirming the more limited effect of ART on immune activation control in the women [8]. Furthermore, in women with HIV infection, the risk of early menopause is one of the possible manifestations of premature aging, as we have also hypothesized in our cohort. From this point of view, it is interesting to note that despite menopause, PLWH remain a neglected area of study and only limited data is currently available to support a relationship between menopause status and immune activation [37]. Evidence from studies of women with autoimmune conditions supports a role for *chronic* inflammation in early menopause onset [38]. Notwithstanding, the current lack of data on a direct relationship between immune activation and early onset of menopause in PLWH increased immune activation in older women living with HIV [39]. The association between HIV and earlier age at menopause were reported. In particular, post-menopausal women with HIV infection receiving antiretroviral treatment and who achieved viral suppression are in a generalized status of immune activation compared to uninfected age-matched controls [40]. Moreover, a cross-sectional analysis from the Canadian HIV Women’s Sexual and Reproductive Health Cohort Study evidenced that 29.7% of women experienced menopause < 45 years: 13.1% with premature menopause and 16.6% with early menopause [41]. Data was confirmed by a study conducted in a cohort of PLWH in Rio de Janeiro, Brazil: 27% of women had early menopause also in this report [42]. Finally, in ANRS CO3 Aquitaine cohort, earlier occurrence of menopause was associated both with HIV-related factors, such as a CD4 cell count < 200 cells/mm, and factors already reported for HIV-negative women (ethnicity and history of injecting drug use) [43]. In summary, our results do not allow us to say with certainty that the patients enrolled experienced an early menopause linked to the state of persistent immune activation; however, a number of evidences in the literature supports the possibility that chronic inflammation and immune activation could be drivers of early menopause also in PLWH.

It is conceivable that multiple and complex molecular, cellular, and endocrine mechanisms act synergistically in determining these sex-related differences between HIV-positive men and women. Early evidence suggests that sex hormones have an important role in modulating variations of innate and adaptive immune responses to HIV infection [7, 44], and our data underline the fact that these differences in immune activation persist also when women experience menopause.

Considering the fact that hormones cannot completely explain the differences in sex-related immune-activation levels, further hypotheses to explain these differences can be proposed, including genetic (sex chromosomes, microRNAs and long non-coding RNA, genetic polymorphisms) and environmental factors (i.e., nutrition, microbiota, physical activity) [24, 44–46].

This recent progress notwithstanding, there is still a profound knowledge gap on the impact of chromosomal effect on HIV-related immune activation, with only preliminary evidence available on the effects of nutritional status, microbiota, and physical activity in modulating inflammation. Therefore, there is still a need for further studies aimed at understanding the causes and minimizing the higher levels of immune activation observed in females with HIV.

Finally, we found that the levels of several immune-activation markers (Tables 3 and 4) differ significantly between males and females in the setting of ART-treated HIV infection, thus suggesting the potential clinical utility in monitoring the pro-inflammatory status of male and female individuals with HIV. Regardless of the pathogenetic implications, this difference could represent a useful tool in personalizing the diagnostic measurement of the state of immune activation by defining suitable cutoffs for each individual based on their sex.

This study has several potential biases: the major limitation of this study is the small sample size and the related statistical concerns. Accordingly, the interpretation of the results must be approached with caution. Moreover, this study suffers from the availability of data only from a single large academic medical center. For these reasons, the study should be considered as a pilot research and larger studies should be performed to confirm and extend these findings.

## Perspectives and significance

Our data suggest that the higher levels of immune activation observed in female ART-treated subjects with HIV may require the preclinical and clinical evaluation of novel therapeutic strategies aimed at better controlling this immune activation. Moreover, the fact that higher immune activation under ART persists in women longer than in men provides a rationale to consider the possibility of treating HIV-positive individuals with different regimens based on their sex.

## Data Availability

Data are available on request to the corresponding author.
